# Cases of Albuminuria

**Published:** 1855-01

**Authors:** F. W. Lewis


### *Cases af Albuminuria.* By F. W. Lewis, M. D.

The subject of Bright's disease of the kidney in its various bearings has been so carefully and thoroughly investigated by a host of distinguished observers, that it would argue a certain amount of presumption in the writer to fill the pages of a Medical Journal with the details of the subjoined cases, in the expectation of eliciting anything very original or new: nor was such his idea in their publication. These cases are merely designed to illustrate and bring forward in a strong light one or two points of interest occurring in connection with a symptom regarded as essential to the disease, viz. dropsical effusion.

No fact is perhaps better established in the history of this singular and protean malady than the liability of dropsical effusions to remissions and exacerbations at longer or shorter intervals, or perhaps of occasional amendments, so considerable and durable that the patient is enabled to attend to his ordinary duties and avocations for months and even years.

The first of these cases is an extreme illustration of this fact, demonstrating as it does, the small prognostic value to be attached to this symptom (dropsy) in Bright's disease, even when in the most advanced and apparently hopeless stage, and also how rapidly and completely it may subside under these circumstances, without any signs of improvement in the disordered eliminatory function of the kidney.

Case I.—Nathaniel Matthews, æt. 45, (colored,) a porter of somewhat intemperate habits, applied for advice at the Philadelphia Dispensary, Nov. 22nd, 1852. He had suffered for about a year with dropsical symptoms and dyspnœa, but up to eleven weeks previous to date of application, he had been able to follow his employment. About this time the dropsy began to increase rapidly, and the difficulty of breathing became so urgent as to threaten suffocation. He was afflicted with an inordinate craving for food, devouring daily enough for two or three adults, and his diet, as is customary with many of the lower classes of negroes during the season of the year when that fish can be procured, consisted mainly of sturgeon fried in oil; a dish which he obstinately persisted in taking during his entire illness, and which there is every reason to believe contributed to hasten his end.

He also complained of heavy lumbar pains, but had noticed nothing abnormal as to appearance or quantity in the urinary discharge.

When I first saw him his face was emaciated, the upper extremities and cellular tissue of the eyelids and chest were slightly infiltrated, while enormous dropsical enlargement of the belly, scrotum and legs existed. The lungs were much compressed by the fluid in the abdominal cavity. The only abnormal auscultatory sign was a moist muco-crepitation, heard posteriorly at the base of both lungs. The heart appeared healthy, and the pulse, though feeble and frequent, regular. Digestion was impaired; thirst tormenting and constant; the appetite as before stated, ravenous, and the bowels usually constipated. The peritoneal cavity was distended with water to its utmost capacity, and the stomach was also filled with gas, circumstances which contributed greatly to the discomfort of the patient by increasing the dyspnœa. His principal suffering, however, he referred to the scrotum. This was so enormously infiltrated as to cause a complete obliteration of the penis, and the urine escaping over the surface had caused inflammation and superficial sloughing of the integuments.

The urine on examination was found to contain albumen, together with the casts peculiar to Bright's disease, in large quantity. The amount passed could not be conveniently ascertained.

The patient, in view of his enfeebled condition, was at once placed on the use of diffusible stimulus (gin and essential oil of juniper) and bitart. of potash given in laxative doses. The scrotum was poulticed and supported.

For the next six weeks the urgency of the symptoms rather increased. The over-distended skin gave way at several points, and a copious furfuraceous eruption resembling pityriasis, attended with acute itching and burning pain, appeared over the abdomen, chest and thighs. The pulse had become very feeble and frequent, at times hardly perceptible ; more than once symptoms of œdema about the glottis were observed, and the signs of pulmonary engorgement were also more noticeable.

Unable to lie down night or day, tormented by restless insomnia and vague hallucinations, presenting occasionally grave nervous symptoms originating in transient attacks of urinary suppression or retention, the patient was rapidly sinking, when as a "dernier ressort," he was tapped at his own request about the latter part of December, and several gallons of fluid drawn off. Contrary to general expectation considerable temporary relief followed, though for a day or two he was alarmingly prostrated. Shortly afterwards, however, the fluid re-accumulated in the peritoneal cavity, and by the end of the next month, January, his condition was nearly as critical as before the operation. For the ensuing three months he dragged on an existence of protracted, hopeless suffering, anxiously and momentarily expecting death, sustained only by stimuli. Twice a large portion of the integuments of the scrotum sloughed away, and vibices formed at various parts of the surface. The body at times exhaled an insupportable ammoniacal odor, and not unfrequently the patient would fall into a comatose condition for days at a time. Various plans of treatment were tried, and all the most valuable and energetic diaphoretics and alterative medicines prescribed separately or in combination. The various preparations of iodine, of potash, arsenic, iron, the vegetable diuretics and hydragogues were pushed as far as prudence would warrant, but nothing afforded the slightest relief. Finally he became dissatisfied, and was induced by his friends to apply elsewhere for medical aid.

For some months I lost sight of him entirely, and towards the end of summer was quite surprised by meeting him on the street carrying a heavy market-basket. Few, if any traces of dropsy were observable, and excepting asthma, an affection to which he had always been liable, he maintained that his general health was altogether re-established. Some drug, administered by a lay-practitioner enjoying an enviable reputation with patients of this class, had, he stated, caused a copious but by no means exhausting drain upon the kidneys and bowels, and a fortnight of this treatment had sufficed to remove the dropsy altogether.

With considerable difficulty a specimen of his urine was obtained and tested. The evidences of progressive disorganization of the kidneys were visible in a notable increase in the amount of albumen and tubular casts. Pus was also detected.

For several months the progress of the case was watched by the writer with considerable interest. The patient during that period had one or two slight relapses, and also wasted in flesh, but by promptly having recourse to the medicine obtained from the lay adviser, (called dropsy pills, and containing certainly gamboge and probably elaterium, in combination with some oily substance,) he was always relieved. During February of the present year, at least 10 months after the disappearance of the dropsy, this symptom recurred, and his system a second time showed evidences of breaking up. The general aspect of the case much resembled that noted during the first attack; the tendency, however, to peritoneal effusion being less, while the disposition to anasarca was greater than at that period.

He found no longer any benefit from using the medicine which had before so frequently relieved him, and I was again requested to attend him. He was placed on the use of iodide of potash, and the gaseous distension of the stomach being a prominent and distressing symptom, carminatives were freely given.

In the course of a fortnight, the anasarca had notedly diminished. The bowels acted more freely, and the urine was copious. From being unable to take food, except in small quantity, his appetite returned, and with it his unhappy predilection for unwholesome articles of diet. For six or eight weeks he continued to improve, and had gained in strength. The fluid had entirely disappeared from the abdomen, and but for the stiffness and cellular induration of the lower extremities, he would have been able to walk about; when, towards the end of July, during the present summer, after an unusually hearty supper of fried fish and cabbage, he fell a victim to his rashness, literally perishing from gaseous distension of the stomach and bowels.

An autopsy was kindly made by Dr. Da Costa in the absence of the writer. Owing to excessive heat and the advanced stage of decomposition of the body, the thoracic viscera were not examined. The liver was healthy, though somewhat enlarged. Both kidneys were found to be twice as large as natural, the external surface was pale yellow and dotted, and a section presented a very characteristic example of the true granular degeneration in an advanced state. Pus existed in the calices, and could be expressed at various points from the substance of the kidney.

The prognosis in any case of albuminuria which presents evidence of organic lesion of the kidney in the tubular casts and organic globules of the urine, has always been and still is regarded as necessarily and uniformly fatal; the above case affords no contradiction to the rule, yet it cannot be denied, that in the present instance the uncertain march of the symptoms and breaks so complete in the chain of morbid action, might tend to mislead the practitioner inexperienced in histological medicine. The presence of albumen alone in the urine has ceased to be regarded as pathognomonic of chronic cachectic nephritis, and in the absence of corroborative proof afforded by this very important aid to diagnosis of the existence of other abnormal elements in the urinary secretion, an observer might reasonably be excused for inferring a favorable termination in such cases.

Another point of interest lies in the very capricious and changing action of medicines at different epochs of the disease, in modifying the amount of dropsical effusion. Science is at a loss for an explanation of these anomalies, which, although they necessarily tend to favor empiricism, yet teach the physician never wholly to abandon hope in any case of dropsy, however desperate in appearance, even after exhausting all the resources of materia medica. A remedy, which at one period faithfully given was ineffectual to relieve, may at another, be productive of signal benefit.

It has been a question, too, as to the advisability of tapping subjects with Bright's disease, when death seems imminent, and more particularly where the ascites is complicated with anasarca. In this connection, the writer may remark that he has twice performed this operation, under these circumstances, with great temporary relief to the patients, the cellular infiltration becoming less, coincidently with the disappearance of the fluid in the abdominal cavity.

The second case affords a striking instance of recovery after the gravest symptoms of constitutional poisoning, from absorption of urea, following urinary suppression.

Hannah Etchel, æt. 21, domestic, was admitted into the Pennsylvania Hospital, (term of Dr. W. E. Pepper,) in the early part of March, 1849, presenting symptoms of general dropsy.

About thirteen years before, while convalescing from scarlatina, she was attacked with anasarca, a condition shortly followed by coma and convulsions. These symptoms apparently produced no lasting impression on her general health, which, with the exception of occasional slight catamenial irregularities, had been quite fair up to the date of the present affection, fixed by her at seventeen or eighteen months before admission to the hospital.

At that period she had measles, succeeded by much the same symptoms as those observed after the scarlet fever, excepting that these, in the last instance, proved more permanent and were associated with, perhaps preceded by, the phenomena of anæmia.

At no time since, had she been entirely free from dropsical effusions; frequent and severe headache, habitual constipation, together with some menstrual disturbance, were complained of during this period.

The condition and symptoms of the patient when I first saw her on the 31st of March, about three weeks after her admission into the medical ward, are described in the following note:

"Body, generally dropsical; cellular tissue of the extremities much infiltrated, and pitting everywhere on pressure; surface cool; complexion waxy, pale; face, and especially eyelids, very puffy; eyes prominent and staring, and both sight and hearing considerably impaired.

Intelligence is average; suffers from constant headache, subject to frequent exacerbations; these last observed to coincide with increase of dropsy, and with more or less embarrassment of the digestion, and urinary function. Patient complains of inward fever and unquenchable thirst; the tongue is red, smooth and pointed; appetite indifferent; there is ordinarily more or less nausea, and sometimes vomiting of light greenish fluid, and obstinate and habitual constipation.

The circulation is languid, pulse averaging 80, feeble; heart's action weak; slight souffle, with the first sound evidently anæmic.

Respiration is healthy; the amount of urine passed varies, being usually abundant, limpid and whey-like, of low sp. gr., precipitating by heat and nitric acid, a copious deposit of albumen."

The treatment at this date consisted in the frequent administration of brisk drastic purges, potass, bitart. and jalap, with infus. of juniper berries, warm bath on alternate nights and a good diet.

A day or two afterwards (April 1st,) an aggravation of all the symptoms took place, probably due to exposure to cold. The headache and nausea became severe and unremitting, the œdema at the same time sensibly gaining ground; her bowels had been unusually confined for some time previously, the jalap, &c. failing to produce a daily evacuation in doubled doses. By the 12th, these symptoms became so urgent, particularly the last, and the dropsical effusion had so considerably increased, that Dr. Pepper decided on prescribing a quarter of a grain of elaterium, to be repeated, if required, in two hours. It is believed, though not positively known, that for three days no discharge from the bowels had occurred; and for the same period, the urine had been passed in exceedingly small quantity. During the day, this drug acted quite freely on the bowels, but there was no reason to infer that any very excessive or debilitating action on the system followed its administration, as in the evening, the patient felt better and more comfortable in every respect, and slept for some hours.

At 3 A. M. was informed by the night nurse that the patient had been seized with convulsions and appeared to be dying. I found her lying on her back on the ward floor in a condition of rigid spasm, and almost without motion; her head thrown back; eyes wide open and staring, pupils dilated and sluggish. Her complexion was ashy pale, and her breathing stertorous; the pulse rather full and frequent. Cups were without loss of time freely applied to the spine, and sinapisms to the epigastrium and extremities. These measures seemed to have the effect of arousing her from the stupor, and of relaxing the spasm somewhat; but whether from the constitutional irritability developed by the application, or from some cause independent of this, she almost immediately lapsed into convulsions, which followed each other at brief intervals up to 6 o'clock, when a gradual subsidence of the symptoms took place. The pulse moderated, stertor disappeared, the pupil contracted to its normal dimensions, and she fell into an uneasy slumber.

At 8 o'clock found the patient sitting up in bed. She was quite rational; complained of intense headache, also of dull, uneasy pains about the muscles of the neck and nucha.

In this condition she remained for about half an hour, when the convulsive seizures recurred with augmented violence. These attacks in all respects resembled the first, with the addition of violent spasmodic twitchings of the facial muscles and rigid upturning of the eyeballs during the paroxysm. The right side of the body was also observed to be more powerfully convulsed.

Soon the cellular tissue of the eyelids, sides of the neck, and upper portion of the chest became greatly swollen, while elsewhere, the effusion was visibly on the increase. Although the pulse was feeble and rapid, the carotid impulse, on the other hand, was quite forcible. During the intervals she was entirely comatose.

At 11 o'clock she was seen by Dr. Pepper, who directed the sinapisms to the spine, and (the patient not being able to swallow) brandy f.℥ss. by injection every hour; but so desperate did her condition appear, and so great the prostration, that but little if any hope was entertained of her surviving till evening.

During the afternoon violent convulsions succeeded each other with brief intervals; the anasarca, meanwhile, rapidly increasing, and the pulse sinking to a mere thread. Partial paralysis of the right side, as indicated by comparative immobility of the arm and leg during the paroxysm, it was thought existed, but this point could not be conclusively established. Inability to swallow continued, but the brandy injections were in great part retained. At 10 o'clock death was momentarily expected to take place. No urine had been passed for 34 hours, during which period the catheter was twice introduced without result.

She lingered in much the same state through the night, the convulsions not being so frequent or violent, but the vital forces being at so low an ebb as to require the hourly administration of an increased amount of brandy by injection. Towards morning the urinary secretion was suddenly restored, a large quantity of water being passed away in bed involuntarily. At the morning visit she presented much the same aspect and symptoms; the twitchings and jactitation were more marked and frequent, the convulsive seizures less so. The condition of the intellect, pupil, and pulse were as before; no alvine evacuation had taken place, and the urine dribbled away uninterruptedly. Ordered injection of ol. terebinth. f℥ss. mixed with the white of two eggs.

About noon a decided abatement in the severity and frequency of the spasm was perceptible. The cutaneous sensibility returned, and the pulse fell to 105. The coma persisted, but was not so complete, and the breathing lost its stertorous character. Great restlessness, with constant tossings and moanings. The pupil began to contract. Later in the day the patient had a free and natural discharge from the bowels, and consciousness in a measure returned. When requested to protrude her tongue, she did so, and with considerable effort managed to swallow a little tea. The ensuing night she passed comfortably, the urine coming away freely, but without her being conscious of the fact.

From this date she began to convalesce, but very gradually. Her first complaint, as consciousness returned, was of loss of sight and defective hearing. For several days her intellect was very dull and confused. She had presence of mind to indicate her wants and regained the control of her sphincters; but when not roused by questions, or by the stimulus of unsatisfied appetite, or natural wants, her condition was one of low delirium. She was the victim of curious and distressing hallucinations, fancied she saw strange objects, such as pigs and cats, around her bed, and, in fact, presented symptoms of cerebral excitement, analogous to those observed in mania-a-potu.

As recovery advanced and the secretions became more regular, the dropsy correspondingly diminished. The headache returned with consciousness, varying in intensity, but always present. The pupils contracting gradually, allowed of a limited and imperfect vision, but there was every reason to infer what subsequently proved to be the case, that grave and irrecoverable lesion of the optic nerve had taken place. With the exception of acute pain and spasmodic contraction of the muscles of the neck and jaws sometimes noticed, convalescence steadily progressed up to the 18th of May, when the œdema slightly increased, more particularly in the eyelids and face. Respiration became hurried and was accompanied by a wheezing sound in the larynx. On ausculting the chest, the left side was found to be dull on percussion posteriorly over lower half, the respiratory sound being scarcely audible over the same extent. She had some cough and viscid expectoration. There was also more or less œdema of the glottis, with infiltration of the uvula and half arches.

A strong solution of argent. nitrat. locally applied relieved this last symptom, but for a day or so the condition of the patient was critical in the extreme. By the 22d she was again convalescent.

She remained in the hospital about a month longer, during which period the amount of albumen in the urine underwent no diminution. The headache lingered, and she still wore the same anæmic aspect: the partial amaurosis and deafness also troubled her somewhat, but, with these exceptions, her general health was much better than it had been for months, and she considered herself as sufficiently re-established to marry, for which purpose she left the institution on the 29th of June of the same year.

Some months afterwards I met her; she had experienced a second attack similar in many respects to the first, only not so severe or so long continued. She was almost childish and helplessly blind. The dropsical symptoms had not been urgent at any time after leaving the hospital. She remained under the care of Dr. B. H. Rand up to the period of her death, which took place in November, 1850, and it is a rather remarkable fact that during these months she complained of little beside the amaurotic affection. She died comatose.

The writer is indebted to Dr. Rand for the following particulars relating to the condition of the body as revealed at the autopsy made by him. The brain was found somewhat engorged (membranes) and there was some serous effusion into the ventricles. The thoracic viscera were healthy, as also were those of the abdominal cavity, with the exception of the kidneys. These were both much enlarged and flabby, of a pale yellow color, presenting a marked example of fatty degeneration.

Suspension of the renal function, an accident occasionally met with in chronic cachectic nephritis is commonly of short duration, and only partial in degree, its effect on the system being manifest in unusual drowsiness and a lethargic condition, amounting at times to coma. Dr. Copland in speaking of the secondary affections of Bright's disease, observes of cerebral complications, that they sometimes occur in the renal malady, but chiefly in its more advanced stage and acute form, and consist of convulsions and apoplectic seizures of longer or shorter duration, and he specially alludes to a mixed state of apoplexy and convulsions as being invariably significant of a fatal termination. The pathological condition found in these cases is commonly effusion of serum containing urea into the ventricles of the brain. That some such condition must have been present in the above case scarcely admits of a doubt, and hence the fact that recovery and reabsorption of serum in the ventricles, or as is not improbable of a coagulum of blood, should have taken place, cannot but be regarded as altogether remarkable, if not unprecedented.

It might, perhaps, be objected that the patient's sex, age, and state of general anæmia, predisposed to convulsive attacks, depending on simple functional disturbance of the brain, but that this could not have been the case is apparent from the undoubted evidence of organic lesion afforded by the sequela.

